# A Curious Problem

**Published:** 1882-06

**Authors:** 


### A CURIOUS PROBLEM.

A correspondent of *Knowledge* having asked how much space would be required on the earth, on the assumption that, beginning with a single pair, the human race had multiplied during 5,000 years at the rate of 30 children to each pair, sons and daughters being born alternately, and each husband and wife being at the time of marriage respectively 21 and 20 years old—there being also no deaths—the Editor replied that the population, taking 30 for each pair at the average age of forty years, would be 2,199,915, followed by 141 digits, which digits, might be represented without appreciable inexactness by ciphers. Now, assuming that ten persons could stand on a surface one yard square, 30,000,000 could stand on a square mile, and on the entire earth, whose surface is about 200,000,000 square miles, some 6,000 millions of millions. Dividing the number just obtained by this, the result is 3,666,526, followed by 125 ciphers. It would require this inconceivable number of worlds like our own for the population on the assumptions made.

Let us see, says Mr. Proctor, how large a single globe would suffice to give standing room to this population, ten to the square yard. Such a globe must have a diameter exceeding the earth's as the cube root of the above quantity exceeds unity, or roughly, as 7 followed by 43 ciphers exceeds unity. (In reality it will be larger considerably, but this is near enough in such a problem as the present.) Now the diameter of the orbit of Neptune, roughly, exceeds the earth's diameter nearly 700,000 times. Hence the diameter of the required globe exceeds the diameter of Neptune's orbit more than a hundred billions of billions of billions of times. Supposing the furthest star visible in the great Rosse telescope to lie some thirteen or fourteen millions of times further from us than the nearest, which lies about 70,000 times further than Neptune, the distance of that star, 1,000,000,000,000 times further than

Neptune (or a light-journey of some 40 millions of years), would be but the 100,000,000,000,000,000,000,000,000th part of the radius of such a globe as would be required to hold the population we are considering. A sphere having a radius equal to 100 millions of years light-journey would not suffice even to contain so many human beings.

